# Effect of melatonin on seed germination of *Cichorium glandulosum* under drought stress

**DOI:** 10.3389/fpls.2026.1947202

**Published:** 2026-09-28

**Authors:** Zhimeng Xu, Zhaokun Wang, Chaoyang Liu, Haichuan Ye, Yan Xia, Yinghao Guo, Jun Yuan

**Affiliations:** 1 Jiangsu Key Laboratory of Regional Specific Resource Pharmaceutical Transformation, Huai’an University, Huai’an, China; 2 National & Local Joint Engineering Research Center for Mineral Salt Deep Utilization, Huai’an University, Huai’an, China

**Keywords:** *Cichorium glandulosum*, drought tolerance, melatonin priming, metabolome, seed germination, transcriptome

## Abstract

*Cichorium glandulosum*, a valuable medicinal herb in Uyghur traditional medicine, mainly reproduces via seeds. Drought stress severely restricts its seed germination and threatens yield stability in the major growing areas. Although melatonin is known to protect seeds against drought in many crops, its roles in this medicinal species remain unclear. In this study, seed priming with 200 μM melatonin was applied to alleviate the adverse effects of drought simulated by 10% PEG-6000, followed by physiological, transcriptomic, and metabolomic analyses. Melatonin significantly improved seed germination performance and seedling growth under drought conditions. Physiologically, melatonin relieved drought-induced oxidative injury and promoted the accumulation of protective substances, while maintaining normal photosynthetic activity. Transcriptomic and metabolomic analyses demonstrated that melatonin reshaped gene expression and metabolic profiles and further improved the overall stress adaptability of plants. We also identified a melatonin-responsive metabolite—fucose—that can independently alleviate drought stress. This study elaborates the holistic regulatory mechanism of melatonin-enhanced drought tolerance across physiological, transcriptional, and metabolic levels. The findings provide a theoretical basis for drought-resistant cultivation and refined field management of medicinal plants in drought environments.

## 1 Introduction

In recent years, drought and water scarcity—exacerbated by climate warming, the intensifying greenhouse effect, and rising environmental temperatures—have become pressing challenges requiring urgent solutions. Drought is a major constraint limiting global crop production (Acosta-Motos et al., 2024). Statistical data indicate that approximately 33% of the world’s arable land is affected by periodic or unpredictable drought (Wicke et al., 2020), particularly in arid and semi-arid regions influenced by global climate change (Suh and Tsheko, 2024). Seed germination is a critical stage in plant growth, and drought stress is one of the most severe abiotic stresses limiting seed emergence (Wicke et al., 2020). Developing strategies to enhance seed survival under drought conditions is essential for mitigating yield losses driven by global climate change. Research has shown that melatonin can alleviate drought stress and promote plant growth in crops such as maize (Ahmad et al., 2022), cotton (Khattak et al., 2022), wheat (Li et al., 2025) and soybean (Liu et al., 2025; Wei et al., 2015). Therefore, applying melatonin to seeds or spraying it during the seedling stage holds promise as an effective strategy to mitigate the impacts of frequent drought, ensure crop emergence, and maintain stable yields.

Melatonin belongs to the class of indole heterocyclic compounds, also known as pineal hormone, melatonin hormone, or melatonin pigment. It is one of the hormones secreted by the pineal gland in the brain. In 1995, scientists firstly discovered melatonin in higher plants, thereby opening a new chapter in understanding its regulatory role in plants. Within plants, melatonin performs multiple functions, including delaying leaf senescence, regulating water balance, promoting lateral root formation, and enhancing seed germination (Kuppusamy et al., 2023). Simultaneously, it scavenges reactive oxygen species, increases antioxidant enzyme activity, and elevates osmotic substance content under drought stress (Sharma et al., 2022). Studies on the molecular mechanisms by which melatonin alleviates drought stress in plants indicate that melatonin, after being perceived by membrane receptors such as PMTR1/CAND2, activates the MAPK cascade through Ca^2+^/ROS signals, thereby regulating transcription factors including DREB/CBF, WRKY, and NAC, and up-regulating the expression of antioxidant enzyme genes, osmolyte biosynthesis genes, and stress-responsive genes (Wei et al., 2018; Sun et al., 2020). Melatonin also cross-talks with hormonal pathways, particularly ABA signaling, by promoting ABA biosynthesis and signal transduction, while modulating auxin, gibberellin, and jasmonic acid pathways to balance growth and stress responses (Li et al., 2015). Furthermore, melatonin enhances the ascorbate–glutathione cycle, photosynthetic systems, flavonoid biosynthesis, and carbohydrate metabolism, thereby maintaining redox and metabolic homeostasis and collectively improving drought tolerance (Cui et al., 2017; Farag-Mohamed et al., 2020; Ren et al., 2025).

*Cichorium glandulosum* is a perennial herb that thrives in warm, dry climates. Its leaves are edible and have been shown to promote bile secretion, act as a diuretic, stimulate appetite, aid digestion, and treat liver diseases (Qin et al., 2019b). Their significant therapeutic effects have been validated in clinical practice (Qin et al., 2019a). In recent years, influenced by global climate change, drought-affected areas have expanded across most of Xinjiang, with particularly significant increases in southern and eastern regions (Wei et al., 2025). As Xinjiang is the primary production area for *Cichorium glandulosum*, this indicates that future yields will be impacted by drought stress. However, current research on melatonin’s effects on Asteraceae plants under drought stress remains limited. Studies have primarily focused on drought resistance in plants like safflower (Heshmati et al., 2021) and chrysanthemum (Luo et al., 2023), leaving a significant gap in research on *Cichorium glandulosum* under drought stress. Therefore, investigating melatonin’s role in enhancing *Cichorium glandulosum* drought resistance is crucial. Such research could significantly improve the plant’s survival rate under drought conditions, thereby boosting its yield, while also enriching the scientific understanding of stress response mechanisms in medicinal plants.

The present study aimed to evaluate the alleviating effects of melatonin on seed germination and seedling growth of *Cichorium glandulosum* under drought stress. By physiological, transcriptomic and metabolomic analyses, we sought to elucidate the underlying mechanisms by which melatonin enhances drought tolerance in this species. This work is expected to provide a theoretical basis for the application of melatonin in improving drought resistance of medicinal plants under water-limited conditions.

## 2 Materials and methods

### 2.1 Plant materials and pharmaceutical reagents

*Cichorium glandulosum Boiss. et Huet.*, belonging to the genus *Cichorium* in the family Asteraceae, was used as the experimental plant material. Seeds of *Cichorium glandulosum* were collected from Yining, Xinjiang, China (approximately 43°55′N, 81°17′E; altitude approximately 640 m) in August 2023. Mature, plump, and intact seeds were selected for the experiments. Melatonin, PEG-6000, proline, indophenol, sulfanilic acid, anthrone, anhydrous glucose, quartz sand, phosphoric acid, Coomassie Brilliant Blue, bovine serum albumin, trichloroacetic acid, thiopental sodium, mercaptoethanol, polyvinylpyrrolidone, methionine, nitroblue tetrazolium, EDTA-2Na, riboflavin, and guaiacol were purchased from Shanghai Yuanye Biotechnology Co., Ltd.; Diethyl ether was purchased from Sinopharm Chemical Reagent Co., Ltd.; Toluene was purchased from Shanghai Jiuyi Chemical Reagent Co., Ltd.; Betaine assay kits were purchased from Beijing Solarbio Science & Technology Co., Ltd.

### 2.2 Experiment design

The experiment was conducted from April to October 2025 at Huai’an University in Huai’an, Jiangsu, China. The *Cichorium glandulosum* seeds used in the experiment were germinated under light conditions in a constant-temperature incubator set at 25 °C and 70% humidity. Seven days after the germination treatment, the seedlings were collected, placed in sealed bags, and stored in a −4 °C freezer for later use.

### 2.3 Establishing drought models and screening for the optimal MT

Based on pre-experiments, drought stress was simulated using 10%PEG-6000. This polymer is widely used to induce drought stress in plants because it lowers water potential to mimic soil drought conditions without being absorbed by plants or causing direct toxicity.

This study employed *Cichorium glandulosum* seeds disinfected with 5% H_2_O_2_. Drought stress was simulated using 10% PEG-6000. Subsequently, seeds were treated with a mixture of 50, 100, 200 and 400 μmol·L^-1^ melatonin and 10% PEG-6000. A 7-day germination experiment was conducted at 25 °C, with a 7-day photoperiod and 70% humidity. Each treatment had 5 replicates, with 40 seeds per petri dish. The germination rate was calculated to determine the optimal MT concentration for seed priming.

### 2.4 Seed priming with MT under drought stress

Germination parameters (germination rate, germination potential, germination index, vigor index), seedling morphology (germ length, radicle length and roughness, fresh and dry weight), and physiological indicators (antioxidant enzyme activity, MDA content, osmotic regulators, photosynthetic pigments) were measured to evaluate melatonin’s effect on *Cichorium glandulosum* seed germination under drought stress.

Processing Settings:①CK: Water-soaked seeds ②DS: 10%PEG-6000+Water-soaked seeds ③DM: 10%PEG-6000 + 200μmol·L^-1^ melatonin-soaked seeds ④MT: 200μmol·L^-1^ melatonin-soaked seeds.

Each treatment had 5 replicates, with 40 seeds per petri dish. The cultivation period was 7 days.

### 2.5 Measurement and methods of physiological indicators

Basic parameter measurements: Germination rate (Hatami et al., 2014), germination potential, germination index, and vigor index (Abdul-Baki and Anderson, 1973) can be calculated using the corresponding formulas.


$$\text{GR}\left(\%\right)=\frac{\text{Total of normally germinated seeds}}{\text{Total number of seeds}}\times 100$$

$$\text{GP}\left(\%\right)=\frac{\text{Number of germinated seeds at a specific early stage}}{\text{Total number of seeds}}\times 100$$

Here, the specific early stage was determined as the 3rd day after sowing (72 hours), at which the number of initially germinated seeds was counted.


$$\text{GI}=\sum_{i=1}^{n}\frac{Gi}{Di}$$

where Gi is the number of germinated seeds on the i-th day, and Di is the corresponding number of days from the start of the experiment.


VI=SGI

Where S is the average seedling length (including roots and shoots) measured 7 days after seed germination.

Germ length, radicle length and roughness were measured using a Vernier caliper. Seedling dry weight and fresh weight were determined by weighing on an analytical balance. Calculate the average value based on 5 replicates.

MDA Content and Antioxidant Enzyme Activity Assays: Malondialdehyde (MDA) was measured according to the thiobarbituric acid method (Zhang et al., 2009). Superoxide dismutase (SOD) was determined according to the nitroblue tetrazolium (NBT) photoreduction method (Wang and Huang, 2015). Peroxidase (POD) was measured according to the Yu-Chang guaiacol method, with absorbance changes monitored at 470 nm (Fleta-Soriano et al., 2017). Catalase (CAT) was determined by UV spectrophotometry according to the H_2_O_2_ decomposition rate at 240 nm (Zhang et al., 2009).

Osmotically active substances were determined as follows: Soluble sugar (SS) were quantified according to the anthrone colorimetric method (Zhang et al., 2006). Soluble protein (SP) was determined according to the Coomassie Brilliant Blue method (Nakano and Asada, 1981). Proline (Pro) was analyzed according to the indophenol colorimetric method (Subramanyam et al., 2019). Betaine (BET) was determined according to the protocol of the betaine content detection kit (Beijing Solarbio Science & Technology Co., Ltd.).

Photosynthetic pigments determination: Photosynthetic pigments were extracted according to the acetone extraction method and calculated according to the formula (Arnon, 1949).

### 2.6 Transcriptome sequencing analysis

Collected *Cichorium glandulosum* seedlings from each treatment group (CK, DS, DM, MT) were immediately frozen in liquid nitrogen and stored at -80 °C for subsequent analysis. Three biological replicates were prepared under each treatment condition, with each replicate composed of seedlings pooled from multiple petri dishes to ensure sufficient sample volume. Total RNA was extracted from approximately 100 mg of frozen tissue using [TRIzol reagent (Invitrogen, USA)] according to the manufacturer’s protocol. RNA purity and concentration were determined using a NanoDrop spectrophotometer (Thermo Fisher Scientific, USA), and RNA integrity was verified via an Agilent 2100 Bioanalyzer. Following total RNA extraction, transcriptome sequencing was performed on an Illumina HiSeq2000 platform (outsourced to YasenDa Biotechnology Co., Ltd.). Gene expression levels were quantified using the Htseq software (v0.6.1) and are reported in FPKM (Fragments Per Kilobase of transcript per Million mapped reads) (Mortazavi et al., 2008). Differentially expressed genes (DEGs) were identified based on a combination of FoldChange and a statistically stringent Q_value (adjusted P_value).

GO (Gene Ontology) enrichment analysis of the DEGs was conducted using the GOseq method (Wang et al., 2012), which employs a Wallenius non-central hypergeometric distribution to correct for gene length bias. KEGG (Kyoto Encyclopedia of Genes and Genomes) pathway enrichment analysis was performed to identify significantly enriched metabolic and signal transduction pathways among the DEGs, using a hypergeometric test against the whole-genome background (Kanehisa and Goto, 2000).

### 2.7 Metabolomics analysis

Collected *Cichorium glandulosum* seedlings from each treatment group (CK, DS, DM, MT) were stored at -80 °C after harvesting. Three biological replicates were taken from each treatment group. Fifty milligrams of freeze-dried, ground tissue from each replicate were extracted using 1.2 mL of 70% methanol solution containing an internal standard. The mixture underwent vortex agitation, ultrasonic treatment, and centrifugation. The supernatant was filtered through a 0.22 μm membrane filter for LC-MS/MS analysis.

Data analysis for metabolomics projects based on an LC-MS/MS system (Orbitrap Exploris 120, Thermo Fisher Scientific, USA) is primarily divided into three parts: basic data analysis, advanced data analysis, and personalized data analysis. Basic data analysis involves performing univariate statistical analysis and multivariate statistical analysis (MVA) on the qualitative and quantitative results of the metabolome to identify significantly differentially expressed metabolites.

Metabolomic analysis of *Cichorium glandulosum* seedlings was performed using a liquid chromatography-mass spectrometry system (Theodoridis et al., 2008; Zhou et al., 2012). Raw data underwent quality control and preprocessing. Multivariate statistical methods, including principal component analysis (PCA) and orthogonal projection to latent structures-discriminant analysis (OPLS-DA), were applied. Differentially expressed metabolites were identified by combining the following criteria: Student’s t-test p-value< 0.05, variable importance in projection (VIP) score > 1 in the OPLS-DA model, and fold change. Metabolites with fold change > 1 were considered upregulated, while those with fold change< 1 were downregulated. Hierarchical clustering analysis, KEGG annotation, and pathway enrichment analysis were subsequently performed on the identified differentially expressed metabolites.

### 2.8 Experimental cultivation of *Cichorium glandulosum* seeds using four metabolites

First, *Cichorium glandulosum* seeds disinfected with 5% H_2_O_2_ were subjected to drought stress simulation using 10% PEG-6000. Subsequently, seeds were treated with a mixture of 10% PEG-6000 and four metabolites(fucose, antipyrine, raffinose, and 2-[[2-(4-hydroxyphenyl)acetyl]amino]glutaric) at varying concentrations. A 7-day germination experiment was conducted at 25 °C, with a 7-day photoperiod and 70% humidity, to assess germination rates. (The germination rate data for four metabolites are included in the **Supplementary Materials**).

## 3 Statistical analysis

Statistical analysis was performed using GraphPad Prism 9.5.0 software. Differences among treatment groups (CK, DS, DM, MT) were assessed using one-way analysis of variance (ANOVA), followed by Tukey’s honestly significant difference (HSD) test or Duncan’s multiple range test. P< 0.05 was considered statistically significant. Data are presented as the mean ± standard error of means (SEM) of biological replicates. Each replicate treatment was treated as an independent experimental unit, and the mean value for each dish was calculated prior to statistical comparison.

## 4 Results

### 4.1 Optimal MT concentration for priming

Under drought stress, the application of different concentrations of melatonin resulted in a trend of initially increasing and then decreasing germination rates in *Cichorium glandulosum* seeds. Compared with the drought-stressed (DS) group, the application of 200 μM MT increased the GR, GP, GI, and VI of *Cichorium glandulosum* seeds by 193.77%, 128.56%, 201.95% and 164.31%, respectively (p<0.05) (**Table 1**). A low concentration of 50 μM MT had virtually no effect on promoting the germination of *Cichorium glandulosum* seeds under drought stress, while the 100 μM and 400 μM concentrations were less effective in promoting seed germination than the 200 μM MT concentration. These results indicate that 200 μM is the optimum concentration for MT priming of *Cichorium glandulosum* seeds. Therefore, 200 μM MT was selected for the subsequent formal priming experiment.

**Table 1:** Effects of different concentrations of melatonin on seed germination of *cichorium glandulosum* under drought stress.

Treatments	Germination rate (%)	Germination potential (%)	Germination index	Vigor index
CK	42.500 ± 1.543^a^	18.357 ± 0.652^a^	12.515 ± 0.498^a^	88.089 ± 3.538^a^
DS	5.714 ± 0.899^d^	2.500 ± 0.546^c^	1.027 ± 0.286^c^	5.497 ± 1.090^c^
DS+50μM MT	6.071 ± 0.922^d^	1.429 ± 0.505^c^	0.191 ± 0.077^d^	0.807 ± 0.336^c^
DS+100μM MT	9.286 ± 0.461^c^	1.786 ± 0.714^c^	0.333 ± 0.141^c^	1.867 ± 0.789^c^
DS+200μM MT	15.826 ± 1.185^b^	5.614 ± 0.481^b^	3.201 ± 0.348^b^	16.567 ± 1.251^b^
DS+400μM MT	10.714 ± 0.714^c^	2.857 ± 1.010^c^	1.464 ± 0.073^c^	10.831 ± 0.542^b^

The data represent the mean of seven replications and the standard error of means (SEM). A one-way analysis of variance (ANOVA) was used to test for significant differences, followed by Tukey’s HSD test for multiple comparisons. Statistical significance was denoted by lowercase letters, indicating that the means with the same letters had no significant difference at p<0.05.

### 4.2 Effect of MT priming on *Cichorium glandulosum* under drought stress

#### 4.2.1 Effect of MT priming on plant growth and photosynthesis under drought stress

Drought stress inhibited the growth and germination of *Cichorium glandulosum* seeds. Under drought stress, the germination rate, germination potential, germination index and vigor index of *Cichorium glandulosum* seeds were significantly lower than those of the control group, decreasing by 87.3%, 90.4%, 83.4% and 93.7%, respectively (**Figures 1B–E**). Following MT treatment, the germination rate, germination potential, germination index, and vigor index of *Cichorium glandulosum* seeds all significantly improved, increasing by 66.0%, 56.3%, 67.5%, and 63.5%, respectively(P<0.05), indicating that exogenous melatonin effectively counteracted the inhibitory effect of osmotic stress on seed germination. Additionally, under drought stress, the fresh weight, germ length, radicle length, and roughness of *Cichorium glandulosum* seeds were lower than the control group, decreasing by 80.6%, 48.4%, 27.7%, and 12.4%, respectively (**Figures 1F, I, J**). Following MT treatment, fresh weight, germ length, and radicle length increased by 56.9%, 10.0%, and 46.8%, respectively, while roughness decreased. Dry weight exhibited abnormal changes (**Figure 1G**), which may suggest altered carbon allocation or water content dynamics under combined drought and MT treatmen.

**Figure 1:**
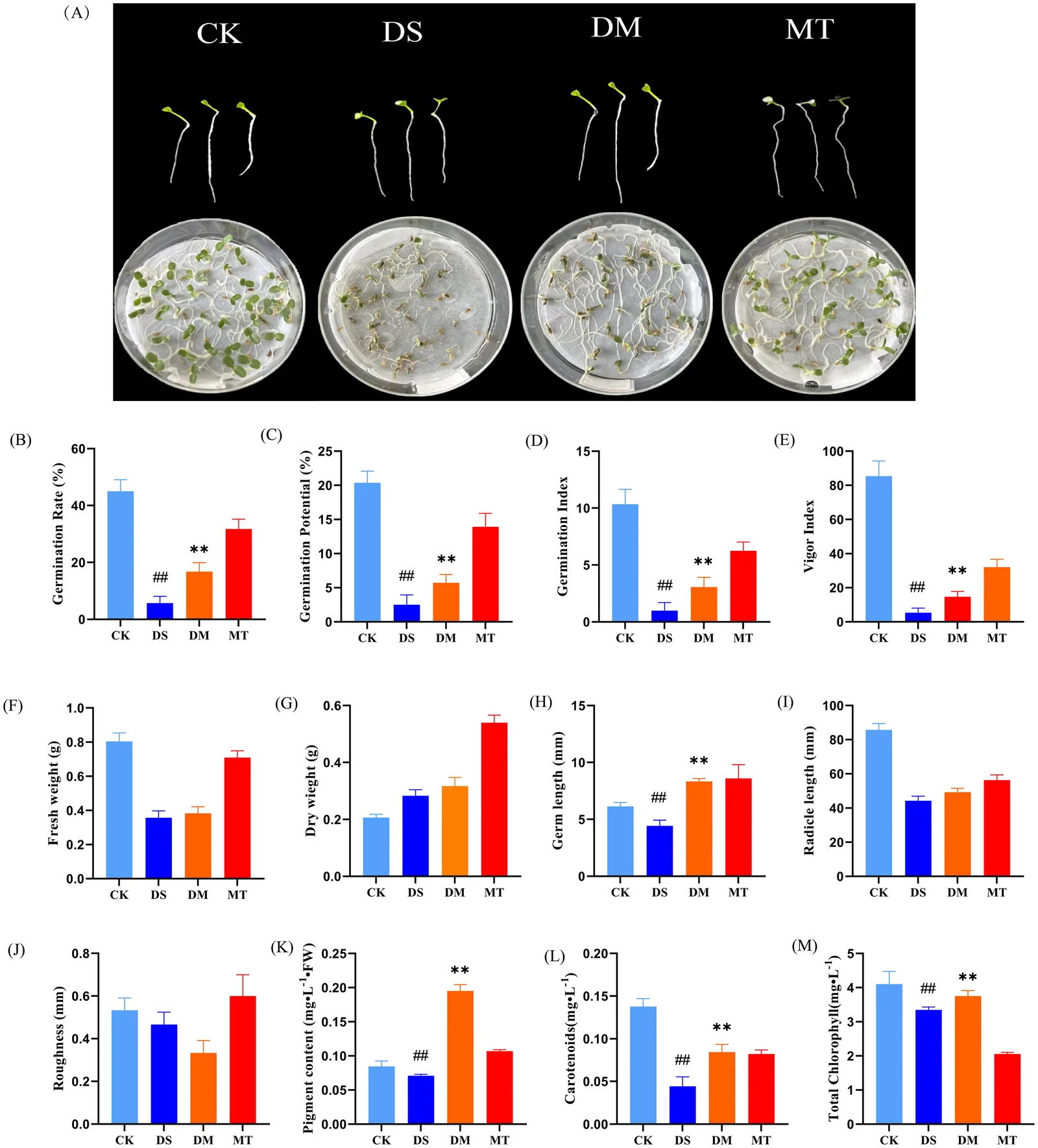
Effect of MT priming on hhenotypic and physiological indicators under Drought Stress. **(A)** Phenotypic traits of germ and radicle of *Cichorium glandulosum* seeds cultured in Petri dish for 7 days. **(B)** Germination rate. **(C)** Germination potential. **(D)** Germination index. **(E)** Vigor index. **(F)** Fresh weight. **(G)** Dry weight. **(H)** Germ length. **(I)** Radicle length. **(J)** Roughness. **(K)** Pigment content. **(L)** Carotenoids. **(M)** Total Chlorophyll. CK: Water-soaked seeds. DS:10%PEG-6000+Water-soaked seeds. DM: 10% PEG-6000 + 200μmol·L^-1^ melatonin-soaked seeds. MT: 200μmol·L^-1^ melatonin-soaked seeds. # indicates a reference material used as a control. * indicates a control material. The data represent the mean of five replications and the standard error of means (SEM). Duncan’s multiple range test was performed, and significant differences are indicated by: **P<0.05.

Under drought stress, the content of pigments content in *Cichorium glandulosum* decreases, but the application of melatonin can increase this content. Compared with the control group, drought-treated *Cichorium glandulosum* seedlings exhibited significantly reduced levels of pigments content, carotenoids, and total chlorophyll, decreasing by 16.2%, 67.8%, and 18.4%, respectively (P<0.05) (**Figures 1K–M**). The combined drought and MT treatment group exhibited significantly higher levels of pigments content, carotenoids, and total chlorophyll compared to the drought-only group, with increases of 170.5%, 47.6%, and 12.1%, respectively (P<0.05). The substantial increase in pigment content indicates that MT may stabilize or promote the synthesis of photosynthetic pigments, thereby maintaining the light-harvesting capacity required for energy production under stress conditions.

#### 4.2.2 Effect of MT priming on osmotically active substances under drought stress

Under drought stress, the content of osmotically active substances in *Cichorium glandulosum* decreases; however, the application of melatonin leads to a significant accumulation of these substances, thereby enhancing drought tolerance. Compared to the CK group, drought-treated *Cichorium glandulosum* seedlings exhibited significantly elevated BET and PRO levels (P<0.05) (**Figures 2A, B**), increasing by 44.6% and 632.4%, respectively. Following the combined drought and MT treatment, BET and PRO levels also significantly increased compared to the drought-treated group, rising by 7.08% and 34.6%, respectively (P<0.05), indicating that MT treatment further enhanced osmotically active substances under drought stress.

**Figure 2:**
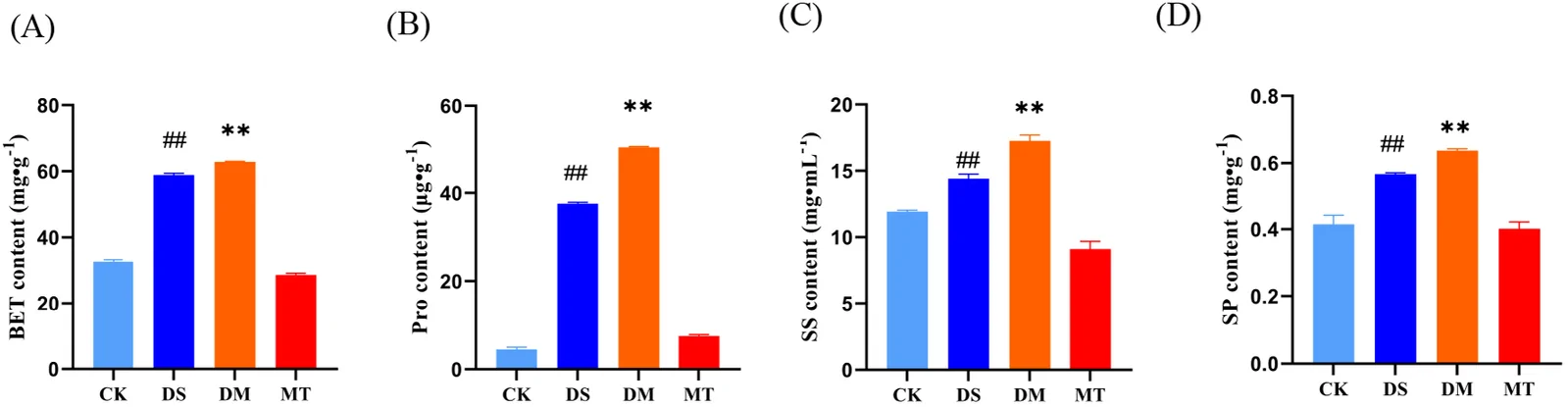
Effect of MT priming on osmotically active substances under drought stress. **(A)** Betaine (BET) content. **(B)** Proline (Pro) content. **(C)** Soluble Sugar (SS) content. **(D)** Soluble Protein (SP) content. CK: Water-soaked seeds. DS:10%PEG-6000+Water-soaked seeds. DM:10%PEG-6000 + 200μmol·L^-1^ melatonin-soaked seeds. MT: 200μmol·L^-1^ melatonin-soaked seeds. # indicates a reference material used as a control. * indicates a control material. The data represent the mean of five replications and the standard error of means (SEM). Duncan’s multiple range test was performed, and significant differences are indicated by: **P<0.05.

Compared with the CK group, the drought-treated *Cichorium glandulosum* seedlings showed a slight increase in SS and SP content, rising by 17.3% and 26.8%, respectively (**Figures 2C, D**). Compared with the drought-treated group, the combined drought and MT treatment significantly increased SS and SP in *Cichorium glandulosum* seedlings, with increases of 16.5% and 11.0%, respectively(P<0.05), which may contribute to maintaining cellular turgor and membrane stability under drought stress.

#### 4.2.3 Effect of MT priming on MDA content and antioxidant enzyme activity under drought stress

Under drought stress, antioxidant enzyme activity in *Cichorium glandulosum* decreases, but it increases following the application of melatonin. The POD, CAT, and SOD activities in the drought-treated group of *Cichorium glandulosum* seedlings were significantly reduced. Compared with the CK group, POD activity decreased by 82.0%, CAT activity decreased by 72.8%, and SOD activity decreased by 33.6% (**Figures 3A–C**), which indicated antioxidant system failure under drought stress. Following combined drought and MT treatment, POD, CAT and SOD activities in *Cichorium glandulosum* seedlings significantly increased compared to the drought-only group, rising by 292.3%, 177.3% and 27.7%, respectively (P<0.05). The particularly strong induction of POD and CAT suggests that melatonin preferentially upregulates the enzymatic pathways responsible for hydrogen peroxide detoxification.

**Figure 3:**
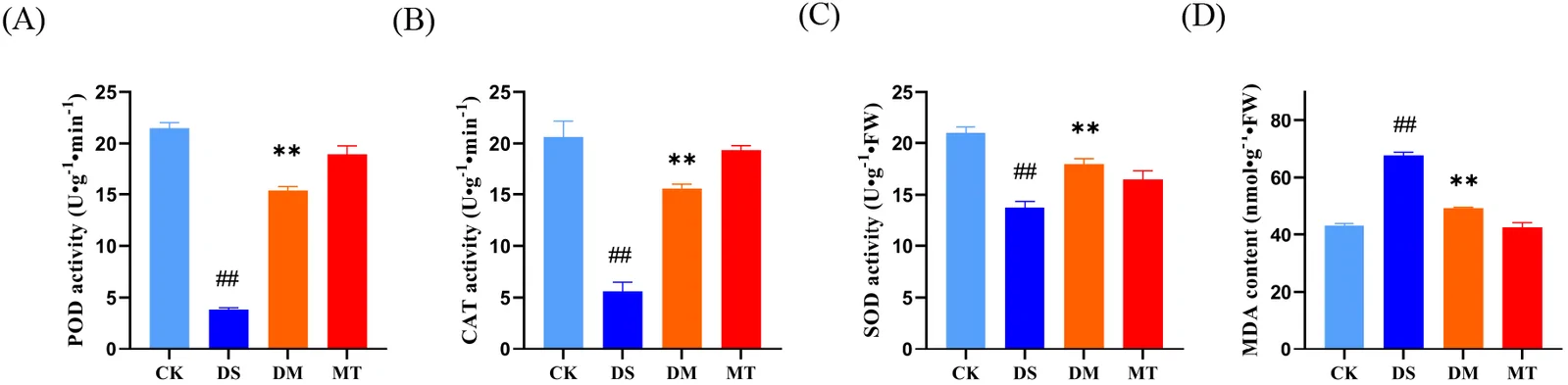
Effect of MT priming on MDA content and antioxidant enzyme activity under drought stress. **(A)** Peroxidase (POD) activity. **(B)** Catalase (CAT) activity. **(C)** Superoxide Dismutase (SOD) activity. **(D)** Malondialdehyde (MDA) content. CK: Water-soaked seeds. DS:10%PEG-6000+Water-soaked seeds. DM:10%PEG-6000 + 200μmol·L^-1^ melatonin-soaked seeds. MT: 200μmol·L^-1^ melatonin-soaked seeds. # indicates a reference material used as a control. * indicates a control material. The data represent the mean of five replications and the standard error of means (SEM).Duncan’s multiple range test was performed, and significant differences are indicated by: **P<0.05.

The MDA content in the drought-treated group was significantly increased by 55.6% compared to the CK group (**Figure 3D**), indicating substantial membrane lipid oxidation. When treated with MT alone, its MDA content showed no significant difference from the CK group. When drought and MT acted synergistically, the MDA content was significantly reduced by 27.6% compared to the drought-treated group (P<0.05), suggesting that MT-activated antioxidant defense effectively alleviated membrane damage.

### 4.3 Identification of DEGs and functional enrichment analysis

To elucidate the molecular mechanisms by which melatonin enhances drought tolerance, *Cichorium glandulosum* seeds were cultured under control (CK), drought stress (DS), and combined drought stress with melatonin treatment (DM) for 7 days, followed by transcriptome sequencing. A total of 2,457 differentially expressed genes (DEGs) were identified between the control and drought-treated groups (CK vs DS), comprising 1,346 up-regulated genes and 1,111 down-regulated genes (**Figure 4A**). Between the control and melatonin-treated group (CK vs MT), 1,718 DEGs were identified, including 1,050 up-regulated genes and 668 down-regulated genes (**Figure 4A**). A total of 1,632 DEGs were identified between the drought and drought-melatonin co-treated groups (DS vs DM), comprising 908 up-regulated genes and 724 down-regulated genes (**Figure 4A**). Additionally, Venn diagram analysis of DEGs among the three groups revealed 137 shared DEGs (**Figure 4B**). Functional analysis of these core DEGs revealed significant enrichment in ABA signaling, MAPK cascades, and redox homeostasis, suggesting melatonin and drought converge on conserved signaling hubs to coordinate adaptive responses. Clustering heatmap analysis showed distinct separation among samples, with replicates from each treatment clustering together, confirming the reliability of the transcriptome data.

**Figure 4:**
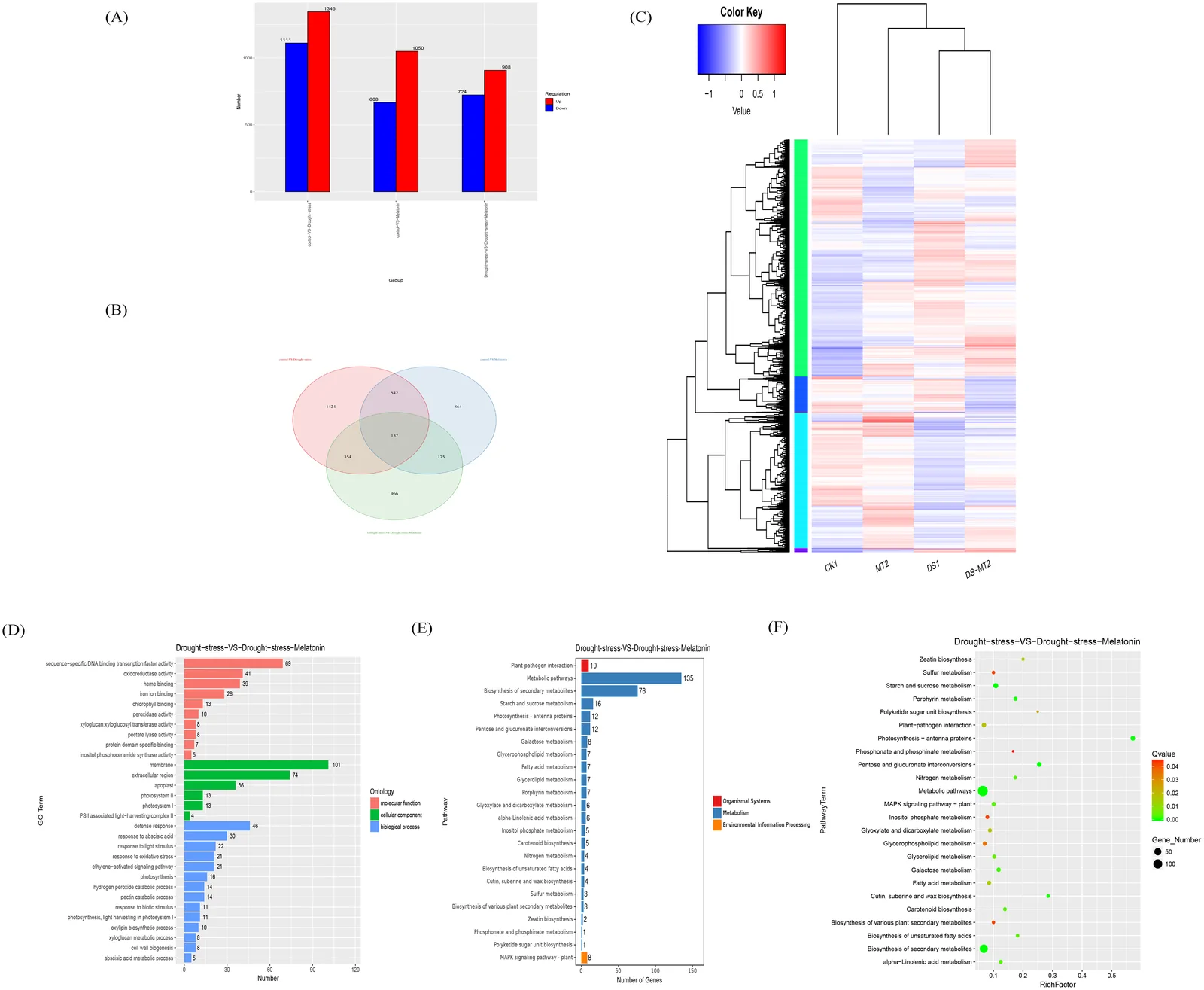
Identification of DEGs and functional enrichment analysis. **(A)** Differentially expressed genes (DEGs) in Cichorium glandulosum under different stress conditions. **(B)** Venn diagram comparing DEGs across groups. **(C)** Clustering heatmap of DEGs. **(D)** Gene Ontology (GO) analysis of DEGs identified from the DS vs DM comparison. **(E)** KEGG analysis of DEGs identified from the DS vs DM comparison. **(F)** KEGG enrichment scatter plot from the DS vs DM comparison.

Transcriptome data were subjected to GO annotation and KEGG enrichment analysis to elucidate the molecular basis of melatonin-mediated drought tolerance in *Cichorium glandulosum* seedlings. Using the comparison between drought-treated and drought-melatonin co-treated groups (DS vs DM) as an example, GO analysis associated the identified DEGs with 30 functional terms, including 14 biological process terms, 10 molecular function terms, and 6 cellular component terms (**Figure 4D**). Notably, among the enriched biological processes, defense response and response to abscisic acid—both critical for plant drought adaptation—exhibited the highest gene counts. This directly links melatonin treatment to the activation of core stress-signaling pathways that regulate stomatal closure and osmotic adjustment. Molecular function analysis revealed significant enrichment in DNA-binding transcription factor activity and oxidoreductase activity, suggesting that melatonin modulates gene expression networks while enhancing cellular reactive oxygen species (ROS) scavenging capacity, a key physiological requirement under drought stress. Among cellular components, plasma membrane and extracellular region were the primary terms associated with DEGs, implicating roles in signal perception and intercellular communication during stress response.

Within organisms, genes coordinate to perform biological functions. Pathway significance enrichment identifies the major biochemical metabolic pathways and signal transduction pathways involving differentially expressed genes. KEGG serves as the primary public database for pathway analysis. In KEGG analysis of the DS vs DM comparison, biological metabolic pathways were categorized into three major groups: organismal systems, metabolism, and environmental information processing. The metabolism category exhibited the highest enrichment, with 331 mapped genes (**Figure 4E**). Notably, biosynthesis of secondary metabolites and biosynthesis of cofactors pathways were most significantly enriched. These pathways directly contribute to physiological drought tolerance by producing osmoprotectants, antioxidants, and stress-protective compounds that maintain cellular homeostasis under water deficit. The enrichment of phenylpropanoid/flavonoid metabolism supports ROS-scavenging compound production, while cofactor biosynthesis provides reducing equivalents for antioxidant enzymes. Thus, melatonin primes metabolism by concurrently activating protective secondary metabolism and redox-cofactor regeneration. Together, these molecular changes—including enhanced ABA signaling, improved ROS detoxification, and elevated secondary metabolite production—collectively underpin the improved physiological performance.

### 4.4 Study on melatonin regulation effects under drought stress based on metabolomics

Based on the VIP values of metabolites in the OPLS-DA prediction, differential metabolites were analyzed to identify the top 20 differential metabolites with VIP values. This study first compared the metabolic profiles between the control group and the drought-stressed model group (CK vs DS). As shown in **Figure 5B**, drought stress induced extensive metabolic reprogramming. The significant upregulation of arachidonic acid suggests drought activates stress-related signaling pathways. Concurrently, the accumulation of flavonoids like quercetin 3,7-di-O-rhamnoside and osmoprotectants like inositol galactoside indicates activation of antioxidant and osmotic defense mechanisms.

**Figure 5:**
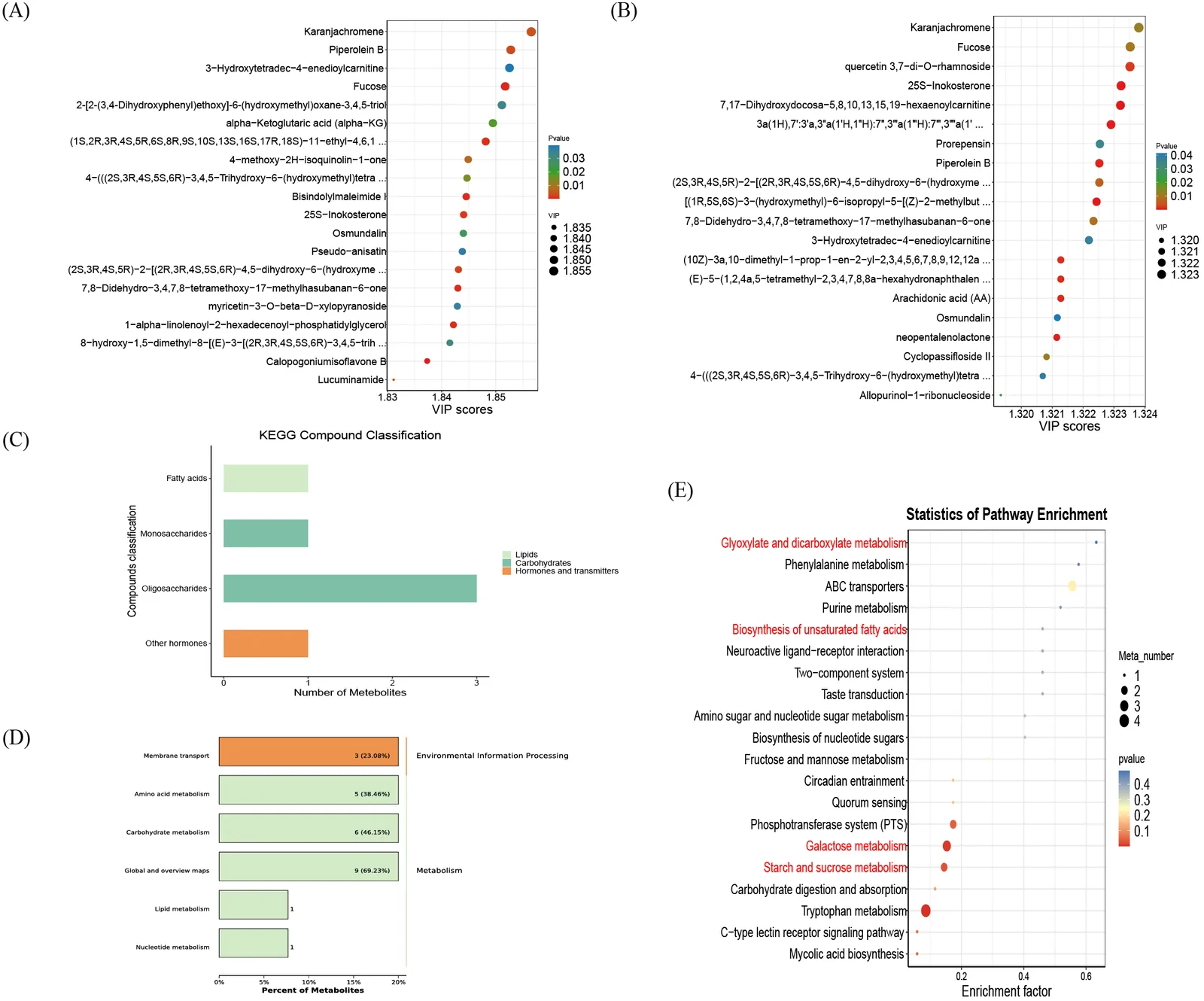
Study on Melatonin Regulation Effects Under Drought Stress Based on Metabolomics. **(A)** Top 20 VIP differential metabolites bubble plot for CK vs DS. **(B)** Top 20 VIP differential metabolites bubble plot for DM vs DS. **(C)** KEGG pathway annotation diagram for DM vs DS. **(D)** Analysis of Pathway Types for DM vs DS. **(E)** Pathway Enrichment Analysis Diagram for DM vs DS.

However, following exogenous melatonin application (DM vs DS, **Figure 5A**), the metabolic profile exhibited a significant favorable shift. First, alpha-Ketoglutaric acid, depleted under drought, was restored after melatonin treatment, suggesting melatonin safeguards energy supply by maintaining TCA cycle stability. This metabolic restoration aligns with the transcriptional induction of mitochondrial carbon metabolism genes, indicating coordinated protection of respiratory ATP generation. Second, melatonin specifically upregulated fucose metabolism, a pathway involved in cell wall repair, enhancing cellular structural integrity against drought-induced mechanical stress.

In the comparison between DM and DS groups, transcription pathway enrichment analysis (**Figure 5F**) and metabolic pathway enrichment analysis (**Figure 5E**) revealed shared metabolic pathways (highlighted in red). These overlapping pathways—including phenylpropanoid biosynthesis, flavonoid biosynthesis, and ABC transporters—demonstrate that melatonin coordinately regulates both transcriptional and metabolic levels to enhance drought tolerance.

### 4.5 Effects of fucose on seed germination of *Cichorium glandulosum* under drought stress

We sorted the results of differential metabolite screening by the absolute value of the Log2Foldchange and selected the top 50 (or all differential metabolites if fewer than 50) for visualization as a bar chart. This process identified several metabolic compounds, including fucose, antipyrine, raffinose, and 2-[[2-(4-hydroxyphenyl)acetyl]amino]glutaric acid. Our study found that the levels of antipyrine, raffinose, and 2-[[2-(4-hydroxyphenyl)acetyl]amino]glutaric acid showed no significant upregulation or downregulation (**Figure 6A**). However, in another group, raffinose levels were upregulated, while antipyrine and 2-[[2-(4-hydroxyphenyl)acetyl]amino]pentanedioic acid decreased (**Figure 6B**). Further analysis is warranted to elucidate the effects of these compounds on drought-stressed *Cichorium glandulosum* seeds.

**Figure 6:**
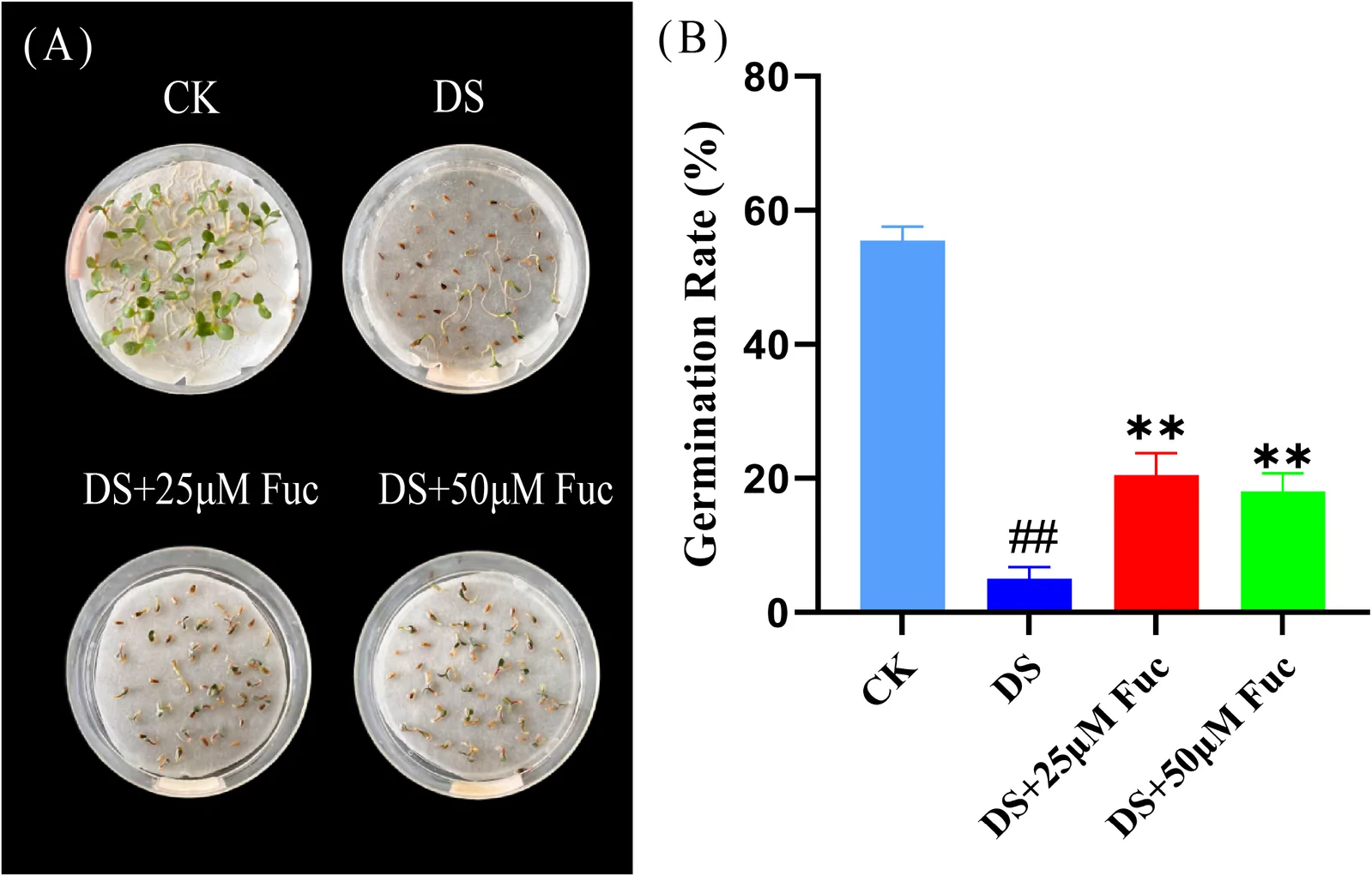
Effects of Fucose on Seed Germination of *Cichorium glandulosum* under Drought Stress. **(A)** Phenotypic characteristics of *Cichorium glandulosum* seedling sprouts cultured in petri dishes for 7 days. **(B)** Germination rate. CK: Water-soaked seeds. DS:10%PEG-6000+Water-soaked seeds. DS+25μM Fuc:10%PEG-6000 + 25μmol·L^-1^ fucose-soaked seeds. DS+50μM Fuc:10%PEG-6000 + 50μmol·L^-1^ fucose-soaked seeds. # indicates a reference material used as a control. * indicates a control material. The data represent the mean of five replications and the standard error of means (SEM). Duncan’s multiple range test was performed, and significant differences are indicated by: **P<0.05.

Based on the data from the list of differentially expressed metabolites (**Table 2**), fucose levels increased sharply under drought conditions in the CKvsDS group. In the DMvsDS group, melatonin treatment led to a slight decrease in fucose levels, which remained higher than normal levels. Therefore, preliminary screening indicates that fucose in *Cichorium glandulosum* seeds can alleviate drought stress. Subsequent studies on metabolite concentration optimization showed that fucose concentrations of 25 μmol·L^−1^ and 50 μmol·L^−1^ increased germination rates by 75.6% and 72.2%, respectively, compared with the drought-treated control group (P< 0.05). The application of fucose may promote seed germination under drought conditions by regulating key metabolic pathways involved in osmotic regulation and reactive oxygen species scavenging.

**Table 2:** Differential metabolites identified in the comparison groups.

Metabolite	RT/min	mz	MeanCK	MeanDS	MeanDM	Comparison	FoldChange	LOG_FoldChange	VIP	Pvalue	Qvalue	signifigamt
Fucose	25.6	187.0571	0.02392	0.58970	0.20170	CKvsDS	24.6543	4.6238	1.3235	0.01034	0.1277	Up
						DMvsDS	2.9234	1.5477	0.5897	0.00091	0.2912	Up

RT, retention time; m/z, mass-to-charge ratio; Mean, mean relative abundance; VIP, variable importance in projection; FC, fold change; log_2_FC, log_2_-transformed fold change.

## 5 Discussion

Water scarcity and drought have severely impacted agricultural productivity. Melatonin, a crucial bioregulator, offers advantages such as low cost, environmental friendliness, and significant efficacy; it can alleviate stress caused by climate change and improve crop quality. Numerous studies indicate that its positive effects in alleviating drought stress are closely linked to its ability to enhance photosynthesis, improve cellular redox balance, and boost antioxidant activity (Cherono et al., 2021; Liang et al., 2019). In our experimental study, drought stress significantly inhibited germination rate, germination potential and germination index of *Cichorium glandulosum*. Subsequent application of melatonin partially alleviated these effects, consistent with previous findings that melatonin mitigates drought stress inhibition in cucumber seeds (Ahammed et al., 2020) and, more recently, in chickpea and alfalfa seeds (Karimian et al., 2025; Zhang et al., 2025). However, in our system, 200 μM melatonin had limited efficacy in improving seed germination rates under severe drought, which is comparable to the concentration-dependent responses reported in lemon (Hosseini et al., 2021) and suggests that species-specific optimization of melatonin dose and application timing is still required. This partial alleviation may be attributed to the relatively short treatment duration or the high drought intensity used here; further experiments with a wider concentration gradient and combined priming agents are warranted.

Drought stress disrupts the structure and function of thylakoid membranes, interferes with the synthesis of photosynthetic pigments and accelerates their degradation (Shaheen et al., 2024), leading to a significant decrease in chlorophyll and carotenoid content in the leaves of *Cichorium glandulosum* seedlings. Exogenous melatonin can effectively alleviate this damage and significantly increase photosynthetic pigment content (Mir et al., 2020; Altaf et al., 2022). Transcriptomic studies have shown that under drought stress, melatonin application upregulates the transcription of photosystem I/II and light-harvesting complex genes (**Figure 4D**), thereby maintaining the structure of the light-harvesting complexes. Simultaneously, it activates porphyrin metabolism and carotenoid biosynthesis (**Figure 4E**) to promote chlorophyll and carotenoid synthesis, resulting in increased photosynthetic pigment levels and thereby conferring drought resistance. These results are consistent with previous findings (Du et al., 2024), which showed that melatonin upregulates chlorophyll biosynthesis genes while simultaneously inhibiting chlorophyll degradation genes, thereby maintaining photosynthetic efficiency under drought stress. Additionally, studies have shown that while melatonin synergistically regulates chlorophyll biosynthesis and catabolism, it also promotes carotenoid cycling, thereby maintaining the stable activity of key photosynthetic enzymes (Arnao et al., 2021). It ensures the stability of photosynthesis by inhibiting pigment degradation and repairing synthetic pathways. This experiment further confirmed that the application of exogenous melatonin promoted the recovery of photosynthetic capacity in *Cichorium glandulosum* seedlings under drought stress by increasing the content of photosynthetic pigments. These photosynthetic protective effects provide the energy foundation for downstream metabolic reprogramming. Building upon this, melatonin further drives the synthesis of osmoprotectants through the regulation of carbon and nitrogen metabolism pathways.

Betaine, proline, soluble sugars, and soluble proteins are crucial osmotically active substances. Their accumulation can alleviate irreversible damage caused by drought, thereby enhancing drought tolerance (Liu et al., 2024). In our experiment, compared with the control group, the levels of these osmotically active substances were significantly higher under drought stress conditions, thereby lowering cell water potential and improving water retention capacity (Cao et al., 2020; Wang et al., 2024). Following melatonin application, the levels of all four osmotically active substances increased significantly. These substances help enhance drought resistance by maintaining cellular hydration and serving as metabolic substrates. Our transcriptomic studies indicated that under drought stress, melatonin treatment significantly upregulated genes associated with starch and sucrose metabolism and galactose metabolism (**Figure 4E**), promoting the synthesis of soluble sugars, and enhanced nitrogen assimilation by upregulating NR, NiR, GS, and GOGAT (Munnik and Vermeer, 2010). These carbon- derived and nitrogen-derived osmolytes act synergistically to lower cellular water potential, stabilize membranes and organelles and improve drought tolerance. Furthermore, the enrichment of the nitrogen metabolism pathway (**Figure 4E**) is closely associated with the synthesis of nitrogen-containing osmoregulators such as proline and betaine. The accumulation mediated by melatonin produces a synergistic effect: while significantly enhancing the cells’ water-retention capacity, it also stabilizes the intracellular membrane system and organelles, thereby improving the drought tolerance of *Cichorium glandulosum* seedlings.

MDA, a product of lipid peroxidation, is commonly used as a marker of oxidative damage to plant cell membranes under abiotic stress (Zhu et al., 2020; Sadak et al., 2020). Drought stress led to a significant increase in MDA levels and a marked decrease in SOD, CAT and POD activities in *Cichorium glandulosum* seedlings; melatonin treatment effectively reduced MDA accumulation while substantially increasing the activities of these antioxidant enzymes, thereby restoring ROS homeostasis (Lee et al., 2024; Li et al., 2025; Wei et al., 2025) and enhancing seedling drought tolerance, consistent with findings in cotton seeds, coffee, and apples (Ahmad et al., 2019; Sharma et al., 2020; Campos et al., 2019). Our transcriptomic data extend these findings by revealing that melatonin also promotes membrane lipid repair: enrichment of glycerophospholipid and glycerolipid metabolism (**Figure 4E**) indicated enhanced synthesis and repair of membrane phospholipids, while activation of alpha-linolenic acid metabolism and unsaturated fatty acid biosynthesis pathways (**Figure 4E**) likely increased the proportion of unsaturated fatty acids through FADs, thereby maintaining membrane fluidity and alleviating lipid peroxidation (Franzoni et al., 2023). At the signaling level, MAPK cascade is enriched. Melatonin may trigger the MAPK cascade via H_2_O_2_ signaling, upregulating transcription factors such as *WRKY1*, *DREB2* and *MYB* (Mashabela et al., 2023), and directly activating the expression of antioxidant enzyme genes such as SOD, POD and CAT, thereby efficiently eliminating ROS. Furthermore, the enrichment of sulfur metabolism (**Figure 4E**) pathways is associated with enhanced glutathione (GSH) synthesis, which further boosts cellular antioxidant capacity (Mansoor et al., 2024). These results indicate that melatonin significantly improved the physiological and biochemical indicators of seed germination and seedling growth under drought stress. Melatonin enhanced cellular stress tolerance through membrane lipid metabolism and phenylpropanoid metabolism, protecting membrane structural integrity and synthesizing secondary antioxidants.

Based on transcriptomic and metabolic pathway analyses, we found that melatonin exerts drought-resistant effects by regulating metabolic pathways; therefore, we proceeded to conduct non-targeted metabolomic analysis. Our metabolomic analysis revealed that exogenous melatonin plays multifaceted roles in metabolically regulating the alleviation of drought stress in *Cichorium glandulosum* seeds, and these metabolic regulatory patterns were closely correlated with the changes in the aforementioned physiological indicators. In the comparison between DM and DS, significant changes in alpha-Ketoglutaric acid (**Figure 5A**) and the enrichment of the glyoxylate and dicarboxylate metabolic pathways (**Figure 5E**) indicate that melatonin effectively maintained the homeostasis of the TCA cycle, providing sufficient energy for photosynthetic recovery and stress responses (Lei and Huang, 2022); The activation of pathways such as starch and sucrose metabolism and galactose metabolism (**Figure 5E**), promoted cell wall polysaccharide synthesis and soluble sugar accumulation, thereby lowering cell water potential and enhancing water retention capacity through osmotic regulation (Niu et al., 2025). At the same time, the differential accumulation of lipid metabolites such as arachidonic acid (**Figure 5B**) and the enrichment of unsaturated fatty acid biosynthesis pathways (**Figure 5E**) indicate that melatonin maintains membrane fluidity by increasing the proportion of unsaturated fatty acids in membrane lipids, thereby alleviating lipid peroxidation (Jannatizadeh et al., 2019; Kong et al., 2020). Moreover, the significant enrichment of the phenylalanine metabolic pathway (**Figure 5B**) and the differential accumulation of flavonoid metabolites such as quercetin-3,7-di-O-rhamnoside and myricetin (**Figure 5E**) indicate that melatonin activates the phenylpropanoid metabolic pathway promotes the synthesis of flavonoid antioxidants. This compound can directly eliminate ROS and protect photosynthetic pigments and membrane structures from oxidative degradation (Xue et al., 2024). The activation of tryptophan metabolism (**Figure 5E**) enhances nitrogen assimilation efficiency. By promoting the synthesis of nitrogen-containing osmoprotectants such as proline and betaine, it works in concert with soluble sugars to stabilize intracellular membrane systems and organelles, ultimately improving the drought tolerance of *Cichorium glandulosum* seeds (Munnik and Vermeer, 2010). In summary, melatonin first maintains photosynthetic structure and energy supply by upregulating photosystem I/II and light-harvesting complex genes and activating porphyrin and carotenoid biosynthesis. It subsequently drives the accumulation of osmoprotectants through transcriptional reprogramming of starch-sucrose metabolism, galactose metabolism, and nitrogen metabolism. Simultaneously, melatonin induces FAD expression and unsaturated fatty acid synthesis to repair membrane lipids, while activating the phenylpropanoid pathway to enhance flavonoid antioxidant production. Ultimately, these four modules converge to activate antioxidant enzymes (SOD, POD, CAT), restoring ROS homeostasis. These modules are mutually supportive at both transcriptional and metabolic levels, collectively constituting a preliminary molecular network through which melatonin alleviates drought stress. In summary, melatonin coordinates physiological, transcriptional, and metabolic responses to maintain photosynthetic structure, osmotic homeostasis, antioxidant defense, and membrane integrity, ultimately promoting Cichorium glandulosum seed germination under drought stress (**Figure 7**).

**Figure 7:**
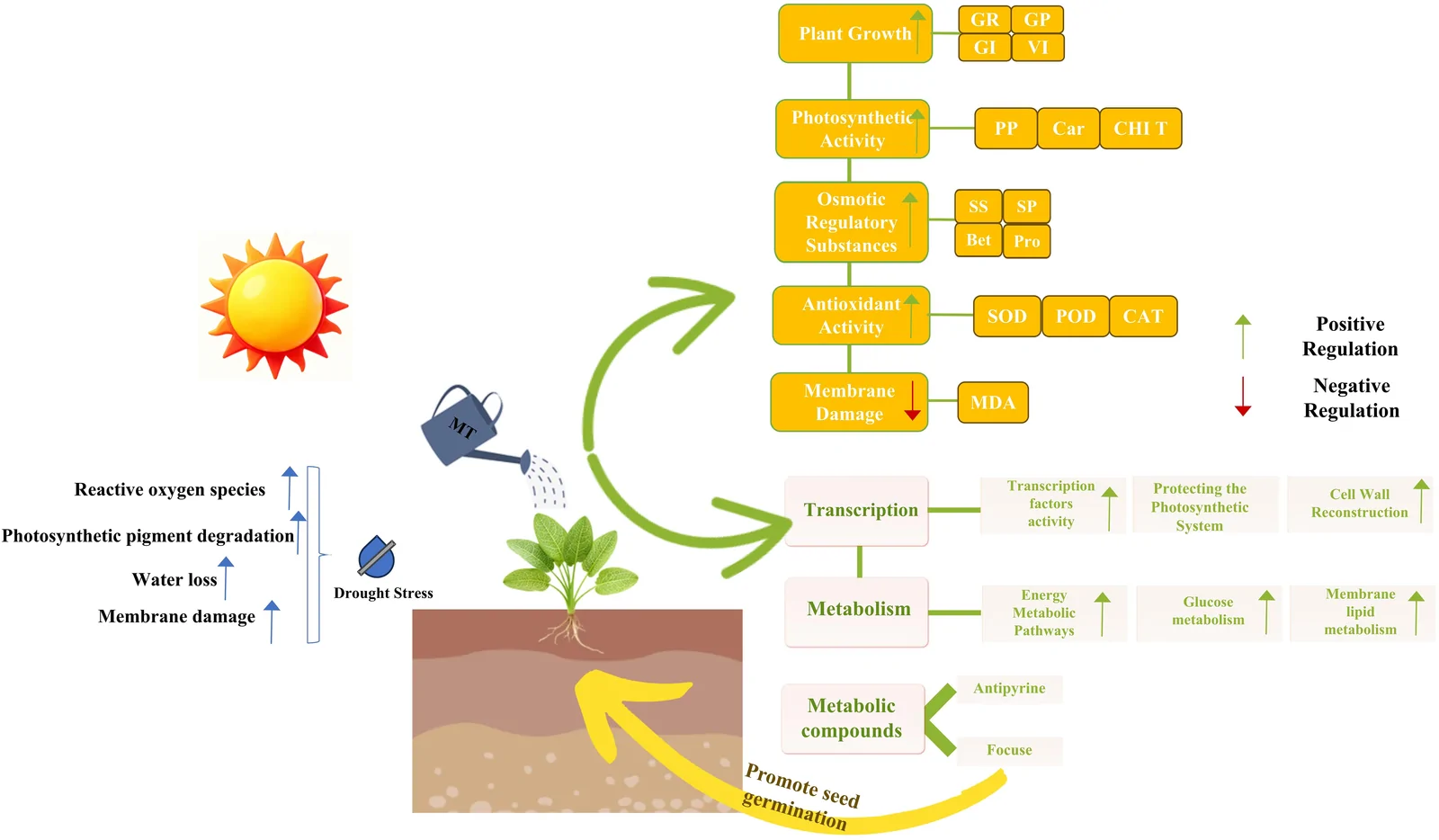
Schematic diagram illustrating the potential mechanism by which melatonin enhances the growth of *Cichorium glandulosum* seedlings under drought stress. GR, Germination Rate; GP, Germination Potential; GI, Germination Index; VI, Vigor Index; PP, Photosynthetic Pigments; Car, Carotenoids; ChI T, Total Chlorophyll; SS, Soluble suga; SP, Soluble protein; Bet, Betaine; Pro, Proline; SOD, Superoxide dismutase; POD, Peroxidase; CAT, Catalase; MDA, Malondialdehyde.

Metabolic studies have revealed that certain metabolites help enhance drought tolerance. Through analysis of changes in metabolite expression levels, we identified four metabolites—fucose, antipyrine, raffinose and 2-[[2-(4-hydroxyphenyl)acetyl]amino]succinic acid—which may have a mitigating effect on *Cichorium glandulosum* seeds subjected to drought stress. However, seed germination experiments indicated that only fucose was able to alleviate the inhibitory effects of drought stress on *Cichorium glandulosum*. As a key component of cell walls (Gamage et al., 2023), fucose administered to *Cichorium glandulosum* under drought conditions may repair seedling cell walls, regulate osmotic control, and counteract drought-induced changes in cell wall tension, thereby mitigating drought stress effects. The metabolite holds promise for application in drought-resistant breeding programs to accelerate the selection of superior varieties. Based on the aforementioned metabolite studies, it has been found that fucose may indeed possess certain drought-resistant effects; however, their more substantial roles require further exploration. Future work should optimize concentrations and delivery methods, and validate these application technologies under field conditions. Ultimately, such research will provide theoretical support for translating laboratory findings into practical applications.

## 6 Conclusion

Exogenous melatonin confers strong drought tolerance to *Cichorium glandulosum* through coordinated regulation of physiological, transcriptional and metabolic processes. It mitigates drought stress damage, stabilizes normal physiological activities and strengthens the overall stress adaptability of seedlings. Our results reveal the multi-dimensional regulatory effects of melatonin on drought response and offer fundamental support for drought-resistant cultivation of this medicinal plant. Further studies are needed to optimize melatonin application protocols and clarify the functions of key responsive substances, which will facilitate the practical application of melatonin in crop stress regulation.

## Data Availability

The original contributions presented in the study are publicly available. This data can be found here: https://www.ncbi.nlm.nih.gov/, PRJNA1533376.
