# Supplementary material for: Effect of melatonin on seed germination of Cichorium glandulosum under drought stress
Source: Front Plant Sci. 2026 Sep 28;17:1947202. doi: 10.3389/fpls.2026.1947202 (PMC13655684; doi:10.3389/fpls.2026.1947202)
Supplement: Supplementary file 1 [file Table1.docx]

Supplementary Material

# 1 Supplementary Data

| Treatments | Germination rate (%) | Germination potential(%) |
| --- | --- | --- |
| DS | 5.00±2.09 | 0.00±0.00 |
| DS+50μM Antipyrine | 9.00±0.61 | 4.50±1.22 |
| DS+50μM Focuse | 9.50±2.29 | 3.50±1.27 |
| DS+50μM Raffinose | 4.00±1.87 | 1.00±0.61 |
| DS+50μM 2-[[2-(4-hydroxyphenyl)acetyl]amino]glutaric | 4.00±1.70 | 0.00±0.00 |

Germination Rate and Germination Potential of Four Metabolites
